# Development of a Subpath Extrusion Tip and Die for Peripheral Inserted Central Catheter Shaft with Multi Lumen

**DOI:** 10.3390/polym13081308

**Published:** 2021-04-16

**Authors:** Han Chang Lee, Jinhyuk Jeong, Seunggi Jo, Dong Yun Choi, Gyu Man Kim, Woojin Kim

**Affiliations:** 1 Safety System R&D Group, Korea Institute of Industrial Technology, 320 Techno-Sunhwan-Ro, Yuga-Myeon, Dalseong-Gun, Daegu 711-880, Korea; hanchang0517@kitech.re.kr (H.C.L.); jinhyuk@vasflex.com (J.J.); tmdrl0723@gmail.com (S.J.); dychoi377@kitech.re.kr (D.Y.C.); 2 School of Mechanical Engineering, Kyungpook National University, #1370 Sangyuk-Dong, Buk-Gu, Daegu 702-701, Korea; 3 Department of Mechanical System Engineering, Kumoh National Institute Technology, Gumi 39177, Korea

**Keywords:** peripherally inserted central venous catheter, multi lumen, polymer extrusion, polymer flow, numerical analysis, viscosity

## Abstract

The tip and die for manufacturing multi-lumen catheter tubes should be designed considering the flow velocity of the molten polymer and the deformation of the final extruded tube. In this study, to manufacture non-circular double-lumen tubes for peripherally inserted central catheters (PICCs), three types of tip and die structures are proposed. The velocity field and swelling effect when the circular tip and die (CTD) are applied, which is the commonly used tip and die structure, are analyzed through numerical calculation. To resolve the wall and rib thickness and ovality issues, the ellipse tip and die (ETD) and sub-path tip and die (STD) were proposed. In addition, based on the results of numerical analysis, the tip and die structures were manufactured and used to perform extrusion. Finally, we manufactured tubes that satisfied the target diameter, ovality, wall, and rib thickness using the newly proposed STD.

## 1. Introduction

A peripherally inserted central catheter (PICC) has the advantage of being able to inject anticancer drugs and contrast agents and collect blood without performing repeated venipuncture, which is a problem for conventional intravenous therapy in supplying fluids, blood, drugs, and nutrition. Joh demonstrated that PICC has a high success rate and relatively low complications. She also noted that it could reduce the issue of central line-associated bloodstream infection (CLABSI) compared to central venous catheters. [1]. Fukuda et al. compared the clinical data before and after CVC and PICC surgery and found that the incidence of PICC complications was very low. Therefore, it is used to inject anticancer drugs or nutritional drugs not only for inpatients but also for cancer patients at home [2]. Yi et al. investigated the factors affecting the discomfort experienced by patients in a general hospital receiving cancer treatment by implanting PICC. As a result, it was found that PICC has the advantage of enabling outpatient treatment, and its use is expanding in an outpatient-centered anticancer treatment environment [3]. Many types of catheters, including PICC, are manufactured with a multi-lumen structure that can play multiple roles in blood vessels with a small inner diameter. They are designed and manufactured in smaller and more complex shapes owing to the advancement of interventional procedures and the demands of clinicians. These multilumen tubes are produced by polymer extrusion, which is suitable for continuous tube manufacturing.

Extrusion is a method of tube manufacturing that applies heat energy and compression energy to a metal or polymer material, which is stretched at a constant speed and then cooled [4]. In particular, the polymer extrusion method applied to a catheter tube begins by melting solid polymer pellets, and then forms the desired shape using a tip and die, which are molds for the creation of tube shapes. At this time, the molten polymer flowing through the tip and die has the property of a non-Newtonian fluid whose viscosity varies with temperature and shear stress, so it is difficult to simply define the flow characteristics [5]. In addition, phenomena such as swelling and melt fracture occur frequently during product production, making it difficult to predict the shape of the final product [6]. In particular, because multi-lumen tubes have much more complex shapes than single lumen tubes, the shapes of the tip and die are complicated, so the discharging rates of the molten polymer flowing through them can be unbalanced. Therefore, designers of tips and dies for the production of high-precision multi-lumen tubes need to consider the rheological properties and fluid dynamic factors of the molten polymers.

Previous studies to optimally design tips and dies have been conducted. Jin et al. studied the complex shape and swelling of the extrudate when designing a polymer extrusion tube with a multilumen profile. They claim that the extrudate shape deforms significantly at the free surface section, which makes the die design and process control very difficult [7]. In addition, Jin et al. analyzed the swelling phenomenon occurring at the tip and die when fabricating a multi-lumen microtube and optimized the air flow rate applied to the lumen. They found that to fabricate tubes with a good shape, the cross-section of the die must be optimized by considering the effect of both the die swell and the gas flow rates [8]. Tian et al. studied microsized double-lumen tube extrusion processes and swelling phenomena using polypropylene. They investigated the change in the swelling effect according to the change in the cross-sectional shape of the tip and die for extrusion, and observed the effect on the diameter of the tube, lumen shape, and thickness of the septum. [9]. Liu et al. used the Taguchi method to analyze the effect of various extrusion process variables on the final extrusion result through numerical analysis and suggested optimal process conditions for the target shape. They found that the most important process variable for extruding a uniform diameter and thickness was the ratio of the winding speed and polymer flow rate (Vp/Fp) [10].

Cho and Lyu compared straight dies and crosshead dies used for catheter tubing extrusion and presented differences in pressure, speed, streamline, shear stress, and residence time according to the die shape. They found that when the pressures at the die inlets were the same, in the case of a straight die the shear stress at the wall of the die land was higher than the critical shear stress, and flow instability may occur [11]. Cho et al. presented experimental data on the characteristics of the extrusion according to the variation of the process parameters and temperatures used for the extrusion of polyether block amide resins. They found that the air pressure, screw speed, and puller speed had the largest effect on the tube size, as in other conventional extrusion processes. Moreover, the distance between the tip and quenching region significantly influenced the ovality of the extruded tube. However, research on tip and die design for manufacturing multi-lumen tubes has not been actively carried out until now. Additional research is also needed on the optimization of the microtube extrusion process with a non-circular lumen, and studies on tips and dies used to stably extrude multi-lumen tubes with an outer diameter of 3 mm or less are required [12]. In particular, in the case of a catheter in which a contrast agent is injected into the lumen using a high pressure of 22 ATM or more, such as PICC, the uniformity of the septal thickness between the lumens and control of the wall thickness of the tube is very important. Therefore, further research on non-circular lumen tube formation should be conducted in depth.

In this study, a new type of extrusion tip and die design model that can stably manufacture non-circular multi-lumen tubes used in PICC was proposed. First, through numerical analysis, the reduction of the swelling phenomenon was verified by comparing and analyzing the velocity field of the molten polymer in the existing tip and die structure and the improved tip and die structure. The proposed tip and die were then manufactured, and used to produce tubes through an extrusion process. Finally, the variations in diameter, ovality, and wall thickness of the tubes produced were investigated according to the change in pulling speed, and the superiority of the newly proposed tip and die was verified.

In this study, three types of tip and die models were proposed and applied:

(1) Circular tip and die (CTD).
(2) Ellipse tip and die (ETD).
(3) Sub path tip and die (STD).

## 2. Materials, Method, and Numerical Modeling

### 2.1. Materials

We used thermoplastic polyurethane Tecoflex EG 85A (Lubrizol Inc., Lakeland Boulevard Wickliffe, OH, USA), which is a biocompatible polymer that is actively used in PICC and other catheter products [13]. The mechanical properties of Tecoflex EG 85A are listed in Table 1 [14].

Extrusion is a processing method that creates a shape by melting the polymer material, so it is necessary to understand the rheological properties in order to analyze the fluid behavior in the tip and die. Therefore, two rheometers were used to measure the viscosity of the molten polymer. Viscosity of the shear rate of 0.1 to 10 s^−1^ was measured with a rotational rheometer (Anton Paar, Inc., Austria, MCR 702 model) [15].

Viscosity in the range of 10 to 10,000 s^−1^ was measured using a capillary rheometer (RG 75 model, GÖTTFERT, Inc., Buchen, Germany). In addition, curve fitting was performed using the Carreau model, as shown in Equation (1) [16,17]:

(1) $$\eta =\eta_{\infty }+\left(\eta_{0}-\eta_{\infty }\right){\left(1+\lambda^{2}\dot{\gamma^{2}}\right)}^{\frac{n-1}{2}}$$

where *η*_∞_ is the viscosity for the infinite shear rate, *η*_0_ is the viscosity for the zero shear rate, λ is the relaxation time, and n is the power-law index.

From the measurement results, it can be observed that the shear viscosity value gradually decreases as the shear rate increases. This result shows a similar trend to the previous studies. [18]. In other words, it was found that the viscosity value of 793.55 Pa·s at a shear rate of 0.1 s^−1^ at 190 °C finally decreased to 58.32 Pa·s as the shear rate increased to 10,000 s^−1^. In addition, it was confirmed that the shear viscosity rapidly decreased in the range from 10 to 100 s^−1^. A similar phenomenon was observed as the temperature increased from 190 to 210 °C. From this, it can be demonstrated that the Tecoflex EG 85A used in the experiment was exhibiting shear thinning as the shear rate increased in the molten state. The viscosity curve fitting data obtained from the measurement results are summarized in Table 2, and the data at 190 °C are reflected in the material data of the numerical analysis.

### 2.2. Design Method

**Figure 1:**
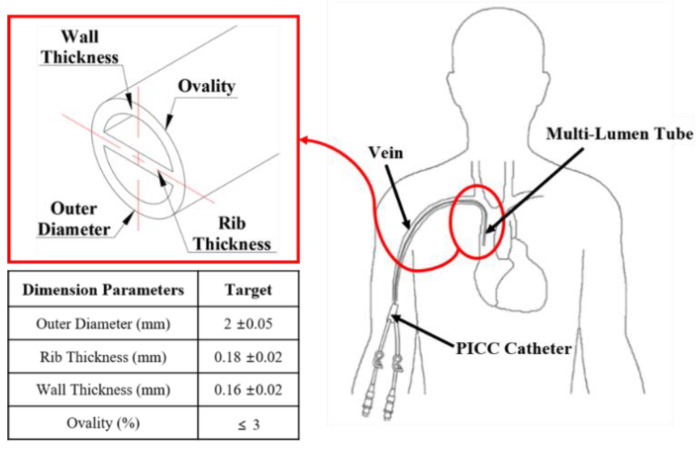
Cross-sectional profile dimensions of target multi lumen tube for peripherally inserted central catheters (PICCs).

As mentioned in the previous section, we aimed to stably extrude the double-lumen shape of the PICC catheter where the uniformity of the thickness of the septum between the lumens is very important, and the shape of the lumen is shown in Figure 1.

The lumen shape shown in Figure 1 is one of the most commonly used tube shapes in use, and detailed dimensions were determined by referring to the dimensions of the products on the market [19]. The outer diameter was designed to be 2 mm (6Fr) considering the blood vessel diameter of an adult, and the two non-circular lumens were designed in a semicircular shape with a cross-sectional area of 1.0 mm^2^ for the inflow and outflow action of blood and contrast injection. The wall thickness where the outer diameter and the lumen are in contact was designed to be 0.16 mm, and the rib shape for dividing each lumen through which blood or drug flows was designed to a thickness of 0.18 mm. [20]. To extrude the target shape, the initial tip and die structures were designed considering the area draw down ratio (ADDR) as shown in Equation (2), which is the ratio of the cross-sectional area between the target product and tip and die. In this study, the ADDR was set to 2, considering previous studies that have proposed ADDR values for industrial polyurethanes [21,22].

(2) $$\mathrm{ADDR}=\frac{D_{d}^{2}-D_{t}^{2}}{D_{o}^{2}-D_{i}^{2}}=\frac{Die\;Area-Tip\;Area}{Outer\;Area-Lumen\;Area}$$

where *D_d_* is the inner diameter of the die, *D_t_* is the outline of the tip, *D_o_* is the diameter of the tube, and *D_i_* is the lumen diameter.

Equation (3) was proposed to quantitatively compare the cross section of a double-lumen tube extruded using a combination of three types of tips and dies. The deformation ratio results were applied to improve the ovality by recognizing the direction and were also applied to the wall/rib thickness to improve the internal shape. In this study, the target range of the deformation ratio for the numerical analysis results was limited to 0.05.

(3) $$\mathrm{Deformation}\;\mathrm{Ratio}\left(\beta_{x},\beta_{y}\right)=\frac{X_{Real}-X_{Target}}{X_{Target}}$$

where $X_{Real}$ is the result of numerical analysis, and $X_{Target}$ is the dimension of the target design.

### 2.3. Numerical Modeling

ANSYS Polyflow^®^ was used to conduct a numerical analysis to predict the flow of the molten polymer at the tip and die and the extrusion result. First, the Navier–Stokes equation was used as the governing equation to numerically simulate the flow of molten polymer generated in the extrusion process, similar to previous research [23]. The flow of the molten polymer was assumed to be an incompressible steady laminar flow with reference to a Reynolds number of 10^−4^ to 10^−2^ in the general polymer extrusion process. In addition, because the flow interval in the process was less than 1 m, we ignored the effects of gravity and inertia. Thus, the continuity and momentum equations are expressed in Equations (4) and (5), as follows:

(4) $$\frac{\partial u_{i}}{\partial x_{i}}=0$$

(5) $$-\frac{\partial p}{\partial x_{i}}+\frac{\partial \tau_{ij}}{\partial x_{i}}=0$$

where $u_{i}$ represents the velocity in the order of i, and p represents the pressure [24]. The stress tensor $\tau_{ij}$ using a generalized Newtonian model is expressed in Equation (6):

(6) $$\tau_{ij}=\eta \left(\dot{\gamma }\right)\left(\frac{\partial u_{i}}{\partial x_{j}}+\frac{\partial u_{j}}{\partial x_{i}}\right)$$

where *η* is the viscosity, and $\dot{\gamma }$ is the shear rate.

**Figure 2:**
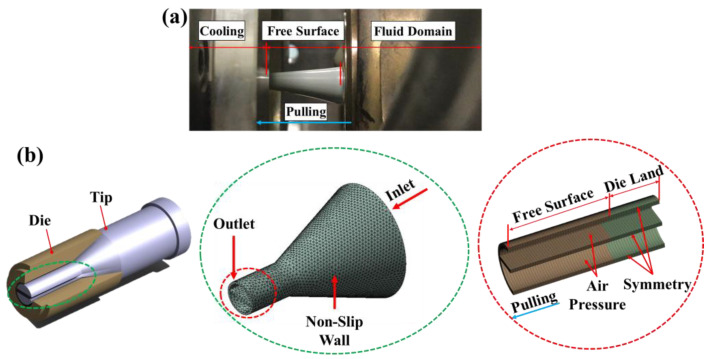
Numerical modeling: (**a**) Photograph of actual extrusion process to be simulated in numerical analysis; and (**b**) image of tip and die, mesh of fluid domain and boundary conditions for numerical analysis.

Figure 2 shows the structure of the tip and die, mesh of the fluid domain for the numerical analysis of the CTD, and the boundary conditions of the analysis model [25]. The fluid domain is composed of the shape of the fluid that flows through the tip, forming the inner shape of the tube while the die forms the outer shape of the tube. The land of the die to obtain the pressure required for stable manufacturing was designed to be 10 mm. We defined the numerical model considering the actual extrusion process and divided it into a fluid domain area where the molten polymer flows and a free-surface area where the final tube shape is formed. Because the shape of the tip and die is symmetrical, only half of the 3D shape (divided vertically) was analyzed to shorten the calculation time. The model was composed of 110,105 nodes and 95,040 elements. For the inlet, a mass flow rate of 100 mm^3^/s was applied. In addition, a non-slip condition was applied to the inner wall of the die and the land section, which is a straight section of the tip. To express the orientation by the puller, a linear velocity of 10 m/min was applied to the end of the free surface [26,27]. Finally, to preserve the lumen shape, compressed air pressure was applied to the lumen wall of the free surface at 2490 Pa. Furthermore, the length of the free surface was set to the same value as that used in the research of Jo and Lee, and the pressure drop of the inlet air was ignored by referring to their conclusions [28].

### 2.4. Experiment Setup

**Figure 3:**
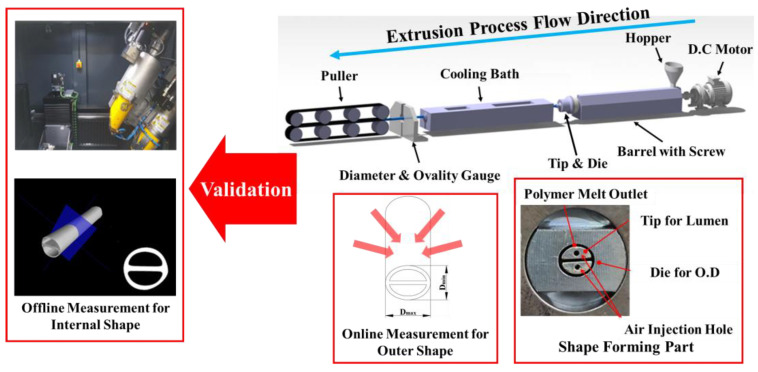
The extrusion system for multi lumen tube manufacturing, diameter/ovality gauge and CT scanner used for validation.

Figure 3 shows the microextrusion system (Davis-Standard Inc., Fulton, NY, USA) used in the experiment. It is a single-screw extruder that is suitable for the production of medical tubes. The screw diameter was 25.4 mm, and the length–diameter ratio (L/D) was 25:1 [29]. The experimental equipment was systemized to electronically adjust the puller speed, screw rotation speed, melting temperature, and air injection, which are factors that can change the cross-sectional dimensions of the extruded tubes. When the molten polymer is discharged to the free surface through the tip and die, the tension at both ends of the extruded tube can be controlled through the puller speed, thereby changing the tube size. The screw rotation speed is also a significant parameter that controls the residence time and flow rate of the polymer supplied through the hopper. As the value increases, the flow rate of the molten polymer increases, so the size of the tube increases, whereas the residence time of the polymer in the tip and die decreases, so the swelling effect can increase dramatically. The melting temperature is a factor for supplying thermal energy that can create a polymer in a molten state that is easy to form. The extruder used in this study was divided into three zones, and we set different temperature ranges for each zone. The barrel in which the polymer was melted by the screw and heater was set at 170 °C. The temperature of the die head where the tip and die were located was set to 190 °C, and the outlet where the molten polymer resin was discharged was set to 190 °C. An appropriate temperature set value was assigned by referring to the recommended process temperatures used in a previous study [14].

Air pressure is a parameter that helps preserve the lumen shape formed by the tip and die until the tube hardens. In this study, to minimize the influence on the pressure fluctuation in an actual extrusion environment, the minimum air pressure (2490 Pa) that can be precisely controlled in the extrusion equipment was applied to the experiment. The process conditions are listed in Table 3.

To measure the quality of the extruded tube, a four-axis laser diameter and ovality gauge (Beta Laser Mike, Inc., Dayton, OH, USA, Accuscan model) was applied to the extruder system as online inspection equipment. The ovality calculation of this measuring equipment follows Equation (7):

(7) $$\mathrm{Ovality}\left(\%\right)=\frac{2\left(D_{max}-D_{min}\right)}{D_{max}+D_{min}}\times 100$$

where $D_{max}$ is the maximum diameter of the tube, $D_{min}$ is the minimum diameter of the tube; that is, when the value of ovality is high, the cross-section of the tube has an elliptical rather than a circular shape.

Using this measuring instrument, the laser interference emitted from up to four directions was read up to 2400 times per second and the instantaneous diameter and ovality values were measured during the extrusion process. Finally, a single layer of the tube was analyzed using an ultra-precision CT scanner (GE Sensing & Inspection Technology, Niskayuna, NY, USA, Vtomex M 240 model), and the shape and inner and outer diameters of each lumen were precisely verified.

## 3. Results and Discussion

### 3.1. Numerical Analysis Result

**Figure 4:**
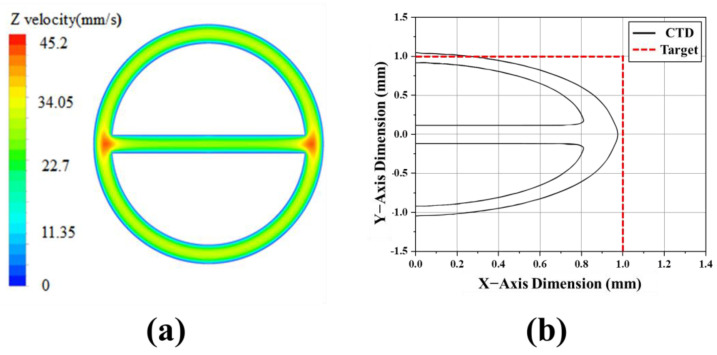
Result of numerical analysis for circular tip and die (CTD): (**a**) Velocity contour at end of tip and die; and (**b**) specification of extrudate tube for measurement of deformation ratio.

Figure 4a shows the velocity field of the molten polymer flowing in the CTD, reflecting the boundary conditions mentioned in the previous section. A maximum flow velocity value of 45.2 mm/s was calculated at the left and right side of the rib and lumen, and the minimum flow velocity of 0 was calculated at the wall surface under the non-slip condition. Based on a numerical analysis, the predicted shape of the extruded product, as shown in Figure 4b, was significantly deformed in the vertical direction compared to the target design shape. This result shows that the swelling force, which is induced by the velocity gradient of the molten polymer and the air pressure acting on the lumen, is influenced by the cross-section of the tube. It was confirmed that the horizontal deformation ratio was −0.05, and the vertical deformation ratio was 0.2. Therefore, in order to address these issues, we proposed a tip and die shaped elliptically in the horizontal direction opposite to the direction in which the deformation occurred.

**Figure 5:**
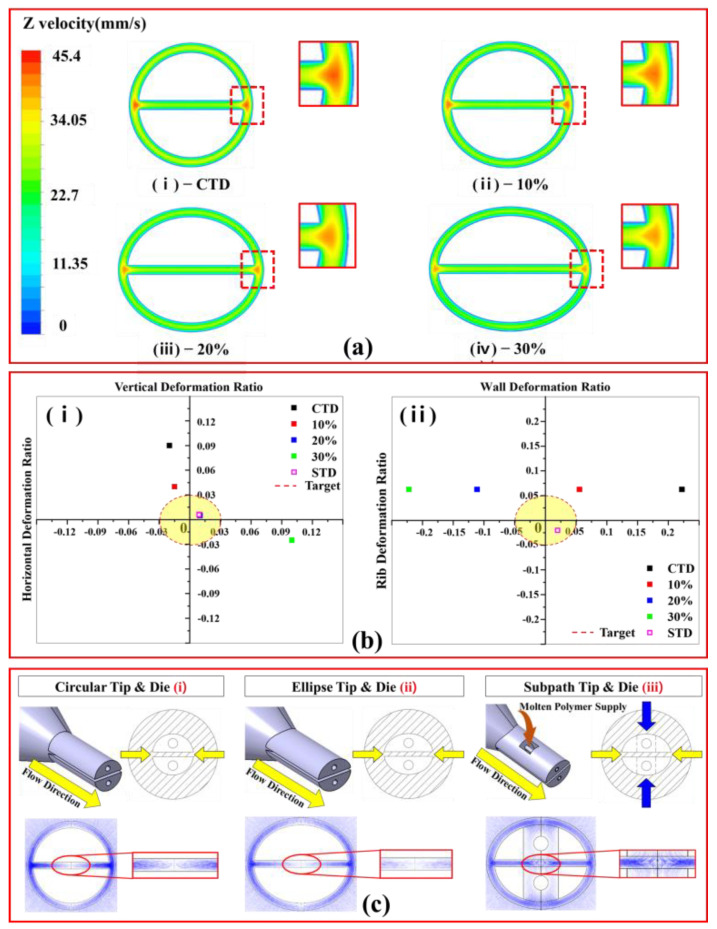
Analysis results using three types of tips and dies: (**a**) Flow velocity contours of various tip and die models; from initial model (**i**) to (**iv**), shape is corrected by 10% to 30% in horizontal direction; (**b**) graph of deformation ratio calculation result for evaluating various tip and die models; and (**c**) schematic diagram of molten polymer supply mechanism for sub path tip and die according to stream line prediction results. Tip and die design improvement procedure: (**a**) Flow velocity distribution of various tip and die models; from initial model (**i**) to (**iv**), shape is corrected by 10% to 30% in horizontal direction; (**b**) graph of deformation ratio calculation result for evaluating various tip and die models; and (**c**) schematic diagram of molten polymer supply mechanism for sub-path tip and die according to stream line prediction results.

Figure 5a shows the cross-sectional velocity fields of the structure correction models of the tip and die designed to improve the ovality. The swelling effect decreased when the tip and die were elliptical in the horizontal direction, and it is believed that the flow velocity of the molten polymer decreased as the outlet area increased.

Based on these results, we calculated the deformation ratio of the vertical and horizontal diameters and rib and wall thicknesses of each design model. The graphs in Figure 5bi shows the distribution of the horizontal/vertical deformation ratio according to the increase in the correction ratio of the tip and die. As the correction rate increased, the vertical direction value decreased.

Among the four analysis models, the results of the 20% model were predicted to have the lowest deformation ratio difference and the lowest ovality value. In the case of the 30% model, the cross-sectional area of the flow path through which the molten polymer passes was increased and the discharge speed was reduced. Thus, the swelling phenomenon was calculated to be the least, but the cross-sectional shape of the extruded tube was calculated to be deformed in the horizontal direction.

These numerical analysis results show that when designing the tip and die to minimize the swelling phenomenon, the correction rate of the tip and die should be designed considering the direction in which the extruded tube is deformed and the air pressure in the lumen. Therefore, in this study, a 20% correction model was selected for the ETD to improve the ovality of tubes extruded through the CTD.

Figure 5bii shows the distribution of the wall and rib thickness deformation ratio as the correction rate of the tip and die increases. This result shows that the wall thickness is larger than the target value in all proposed models. For the rib thickness, the deformation ratio tends to be negative. In particular, the rib thickness deformation ratio after applying the ETD for ovality improvement was calculated as −0.11%. These results indicate that the rib is extruded thinner as the tip and die correction rate increases. To analyze this problem, we analyzed the stream line of CTD and ETD, as shown in Figure 5(ci,cii) and confirmed that the supply of molten polymer to the rib in the ETD is significantly lower than that in the CTD.

These results show that the polymer was not stably supplied to the center of the tip where the rib of the tube was formed, because the ETD’s cross-sectional area was designed to be larger than the CTD under the same flow rate and pulling conditions, so the discharge speed was relatively slow.

Therefore, to solve this problem, we designed a sub-path that can supply the polymer to the center of the tip, and compared the streamline using three tip and die design models, as shown in Figure 5c.

As a result, unlike the result of ETD in which the unstable flow of molten polymer was observed compared to CTD, in the calculation result applying STD, polymer flow was observed at the center of the tip.

Therefore, a sub-path was designed to increase the rib thickness. It was designed to supply the molten polymer actively to the center of the tip. To verify the performance of the new design model, streamlines of the molten polymer inside the CTD, ETD, and STD were compared, as shown in Figure 5c.

Additional molten polymer was supplied through the sub-path, and it was verified that more streamlines were formed than in the case of ETD, so the ribs would be reinforced. Moreover, through the deformation ratio distribution analysis, we also confirmed that STD is advantageous in manufacturing tubes with improved ovality in a similar way to ETD, and also helps to increase the thickness of the ribs between the lumens. Therefore, the STD was proposed as the optimal design model.

### 3.2. Experiment Result

**Figure 6:**
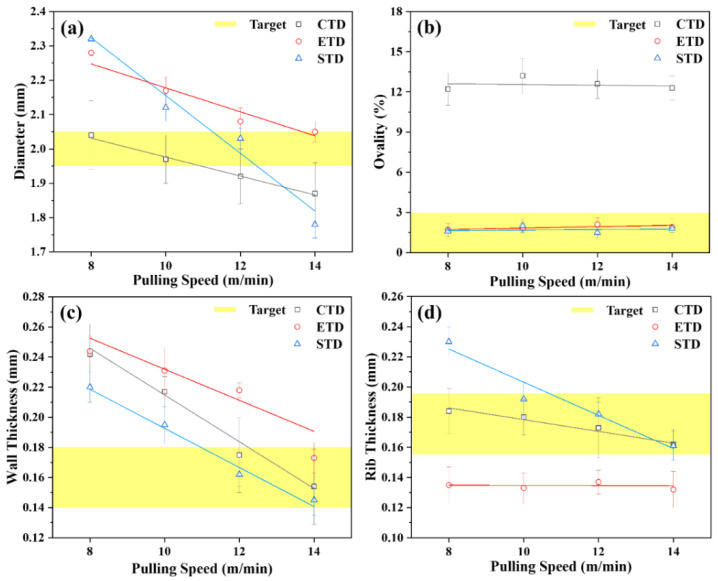
Measurement result of various dimensional parameters according to variable pulling speed for each design model: (**a**) Average outer diameter; (**b**) ovality; (**c**) wall thickness; and (**d**) rib thickness.

Based on the numerical analysis, we designed and manufactured CTD, ETD, and STD and conducted an experiment to verify the design and numerical analysis. All three types of tip were made of STS420 with strong corrosion resistance and good thermal conductivity. For precise shape processing, the cylindrical body of the tip was machined with a CNC lathe, and the land part was wire-discharged to combine the two parts. In addition, the inlet of compressed air for preserving the shape of the lumen was wire-discharged to 0.78 mm. In the case of the die, the entire shape was machined with a CNC lathe, and the inner shape was wire-discharged to form an inner diameter of 4 mm for the initial model and an ellipse type of 5.84 × 5.45 mm^2^ with a 20% shape correction. The graph in Figure 6a shows the change in the outer diameter of the tubes by varying the pulling speed of the three types of tip and die models at the same flow rate and air pressure. In all experimental groups, as the pulling speed increased, the overall outer diameters of the tubes decreased. In addition, when three types of tip and die model were applied, the target outer diameter could be manufactured. However, when using CTD, the ovality value was the highest, and the error range of the outer diameter was wide.

Figure 6b shows the ovality variations according to the pulling speed. As calculated by numerical analysis, the ovality was the highest in all experimental groups of CTD. In the experimental groups when ETD and STD were applied, ovality was distributed within a 3% error range. The graph in Figure 6c shows the measurement results for the wall thickness. The thickness decreased with increasing pulling speed, similar to the previous outer diameter results. In particular, when comparing the ETD and STD results, it was observed that the wall thickness of the STD experimental group was thinner than that of the ETD. This phenomenon indicates that some of the molten polymer was fed into the ribs between the lumens through the sub-path to form the walls.

The graph in Figure 6d shows the variation in rib thickness according to the pulling speed. The results in the CTD and STD cases showed that the thickness of the rib decreased with increasing pulling speed, and demonstrated that sufficient molten polymer was supplied to all areas of the tip and die. However, when ETD was applied, the thickness of the ribs in the tube did not change even when the pulling speed was changed, because the molten polymer did not feed smoothly into the center of the tip, as calculated in the numerical analysis. To summarize the experimental results, when STD was applied at a pulling speed of 12 m/min, the outer diameter, ovality, wall thickness, and rib thickness all satisfied the target.

### 3.3. Discussion

#### 3.3.1. Comparison of Numerical Analysis and Experimental Results

The numerical and experimental results under the same conditions are summarized in Table 4 to verify the reliability of the numerical analysis performed in this study.

In comparing the experimental and numerical analysis results for the CTD-applied tube, the difference in Dmax was not large, but the *D_min_* value was larger in the actual extrusion result. This means that the ovality of the tube manufactured in the actual extrusion environment was higher than the calculated value. In the comparison results for the EDT-applied tube, the values of the diameter and wall thickness are larger in the actual results than in the analysis. This means that the supply of the molten polymer to the center of the tip was unstable in the actual extrusion environment. The results for the EDT-applied tube showed most specifications larger in the experimental results than in the numerical analysis. However, considering the operating conditions and environmental conditions in which the extruder operated, the trends of the numerical analysis results were judged to be reliable.

**Figure 7:**
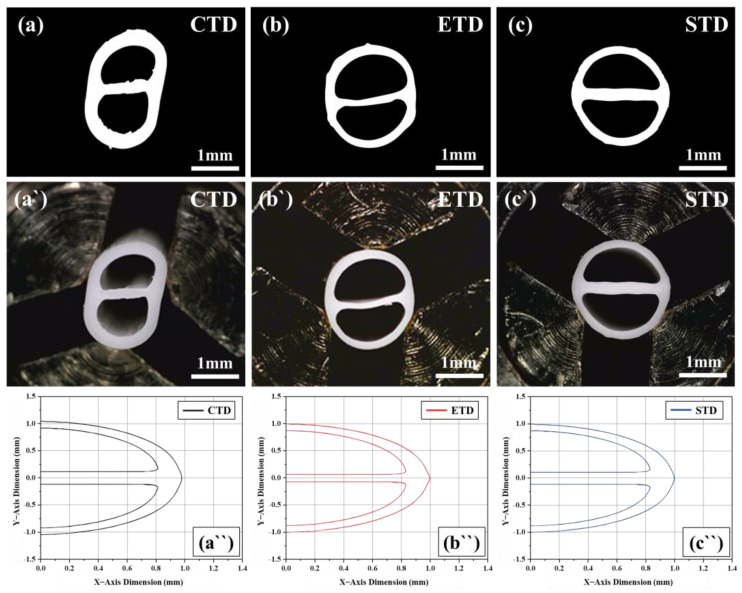
Comparison of numerical analysis and experimental result: (**a**–**c**) 2D tomographic images; (**a′**–**c′**) photograph of actual multi lumen tubes; and (**a′′**–**c′′**) wire frame data of numerical analysis result.

Figure 7 shows the shape of the multi-lumen tubes manufactured under the same process conditions as the numerical analysis, comparing 2D tomographic images through a CT scanner (a, b, c), actual tube photographs (a′, b′, c′), and the wire frame data of the numerical analysis results (a′′, b′′, c′′). Although the numerical analysis did not calculate the dimensions of the extruded tube, the vertical deformation phenomenon when applying CTD (a, a′, a′′) resulted in similar results between the numerical analysis and the actual experiment. The phenomenon of thin extruding rib thickness that occurred in the results for ETD (b, b′, b′′) was the same in the numerical analysis and the actual experiment results. These results verified that the direction of deformation can be predicted in the numerical analysis step by analyzing the velocity field for the cross-section. In addition, by analyzing the streamline, it was demonstrated that the flow condition can influence the extrusion result. In future work, based on this research, it will be necessary for produce calculations similar to actual results by adding material data that can better express viscoelasticity and more accurate meshing technology.

#### 3.3.2. Comparison of Multi Lumen Tubes

**Figure 8:**
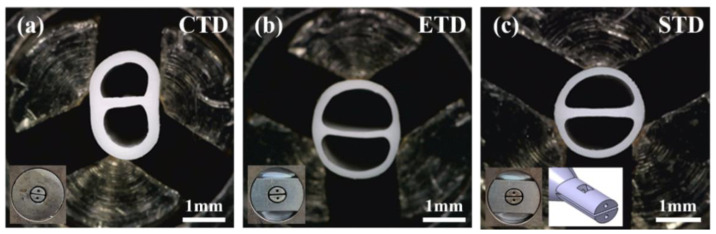
Comparison of multi-lumen tubes produced under same process conditions (pulling speed: 12 m/min): (**a**) CTD; (**b**) ETD; and (**c**) STD.

Figure 8 shows the multilumen tubes manufactured in each tip and die model. The multi-lumen tube was manufactured with a pulling speed of 12 m/min, which is a condition producing results similar to the target value when STD is applied. Detailed specifications are summarized in Table 5.

First, the cross-sectional results of the extruded tubes using CTD and ETD were compared. The ovality of the CDT-applied tube was 12.6%, while the ETD-applied tube was significantly improved to 2.1%. This result is similar to the trend observed in the numerical analysis results. In comparing the ETD-applied tube and the STD-applied tube, the ovality of the tubes was 2.1% (ETD case) and 1.5% (STD case), respectively, and all tubes were within the target range. This is because the shape correction rates of the ETD and STD are the same at 20%. In addition, the tube to which STD was applied had a rib thickness of 0.182 mm, which was closer to the target shape than that of the ETD (0.132 mm). From these experimental results, we demonstrated that the tip and die using the sub-path are effective in simultaneously improving the ovality and rib thickness of the tube.

## 4. Conclusions

We developed an optimal tip and die for the production of a peripherally inserted central catheter (PICC) shaft with a multi-lumen. To implement the target specification, we designed an initial model and analyzed it using a numerical analysis. The velocity distribution of the molten polymer on the tip and die sections and the swelling effect were numerically investigated. Moreover, a circular tip and die (CTD), ellipse tip and die (ETD), and sub-path tip and die (STD) were proposed and manufactured to verify the tubing extrusion performance and reliability of the numerical analysis. The conclusions of this study are as follows.

1. Through numerical analysis, the deformation of the tube due to the flow velocity imbalance of the molten polymer in the tip and die was calculated, and a novel tip and die structure was proposed to resolve this problem. In the actual extrusion process, when the swelling force applied to the molten polymer and air pressures are stably balanced, a tube with low ovality can be formed.
2. As the cross-sectional area of the tip and die increased, the flow velocity decreased and the internal path of the polymer increased, resulting in a lack of supply to the center of the tip. To solve this problem, a structure in which a sub-path was added to the tip was proposed, and the stability of the flow of the molten polymer was numerically validated. It was confirmed that the tube manufactured through the extrusion process also showed similar performance to the numerical analysis results.
3. Based on the above research results, a tip and die design method which can control the ovality, rib and wall thickness simultaneously is proposed.

In this study, we focused on the effects of the tip and die structures of multi-lumen tubes. In future work, we plan to analyze the flow characteristics of various molten polymer materials and apply them to the production of multi-lumen tubes of various shapes. In addition, for numerical analysis, viscoelastic fluid analysis, and inverse problems will be applied to obtain clearer analysis results.

## Figures and Tables

**Table 1:** Mechanical property of Tecoflex EG 84A.

Mechanical Properties	Unit	Value
Hardness	Shore A	85
Density	kg/m^3^	1250
Melting Point	°C	180
Flexural Modulus	MPa	18.6
Tensile Stress at Break	MPa	38.9

**Table 2:** Shear viscosity parameters in Carreau model after curve fitting.

Temperature [°C]	Fitting Parameters			
	$\eta_{0}$ [Pa·s]	$\eta_{\infty }$ [Pa·s]	λ [s]	*n*
190	655.89	1.91 × 10^−6^	0.0089	0.6
200	183.83	1.85 × 10^−5^	0.002	0.65
210	74.7	2.45 × 10^−5^	0.001	0.7

**Table 3:** Experimental factors for evaluation of each design model.

Test No.	Screw Speed (rpm)	Air Pressure (Pa)	Pulling Speed (m/min)
1	10	2490	8
2			10
3			12
4			14

**Table 4:** Comparison of numerical analysis and experimental results under similar process conditions.

Dimension	Tip and Die Type						Unit
	Circular (CTD)		Ellipse (ETD)		Subpath (STD)		
	Analysis	Experiment	Analysis	Experiment	Analysis	Experiment	
*D_max_*	2.15	2.14 ± 0.07	2.02	2.20 ± 0.04	2.00	2.20 ± 0.03	mm
*D_min_*	1.95	1.87 ± 0.07	1.99	2.16 ± 0.03	1.98	2.18 ± 0.03	
Rib Thickness	0.221	0.182 ± 0.012	0.163	0.133 ± 0.01	0.181	0.192 ± 0.012	
Wall Thickness	0.151	0.217 ± 0.01	0.152	0.231 ± 0.015	0.152	0.198 ± 0.012	
Ovality	9.75	13.47	1.50	1.83	1.01	0.91	%

**Table 5:** Dimensional specifications of multi lumen tubes made by different tip and die models.

Dimension	Tip and Die Type				Unit
	Target Model	Circular (CTD)	Ellipse (ETD)	Subpath (STD)	
Diameter	2.00 ± 0.05	1.92 ± 0.08	2.08 ± 0.04	2.03 ± 0.03	mm
Rib Thickness	0.18 ± 0.02	0.183 ± 0.02	0.132 ± 0.008	0.182 ± 0.008	
Wall Thickness	0.16 ± 0.02	0.155 ± 0.025	0.173 ± 0.005	0.161 ± 0.005	
Ovality	≤3	12.6 ± 1.1	2.1 ±0.5	1.5 ± 0.4	%

## Data Availability

Not applicable.
